# Teaching research systematically: From educational policy vision to competence-oriented practice

**DOI:** 10.3205/zma001779

**Published:** 2025-09-15

**Authors:** Sören Moritz, Christoph Stosch

**Affiliations:** 1 University of Cologne, Faculty of Medicine, Dean’s Office, Vice Dean for Teaching and Studies, Cologne, Germany; 2 University of Cologne, Faculty of Medicine, Dean’s Office, Vice Dean for Research – Translation – Transfer, Cologne, Germany

## Editorial

The systematic teaching of scientific competencies is a central prerequisite for enabling future physicians to make evidence-based decisions, think critically, and continue lifelong learning. The National Competency-Based Learning Objectives Catalog for Medicine (NKLM) explicitly calls for integrating scientific thinking and working methods into medical education [1]. The German Council of Science and Humanities also emphasizes the need to introduce students to scientific methods and research at an early stage [2].

In German higher education didactics, this is often referred to as "research-based learning." Developed in the context of the reform movements of the 1970s, the approach shaped by Ludwig Huber undoubtedly represented a significant educational policy impetus. His emphasis on the student as an active subject and his questioning of the traditional lecture culture are undisputed achievements [3]. However, in practice, this approach was often reduced to the demand for as much unguided research as possible. The underlying assumption that science is learned primarily through autonomous action has proven insufficient from today’s perspective. Scientific work is not an innate ability but a highly complex competence that must be taught systematically and structurally. Empirical studies show that open learning formats without didactic support regularly lead to overload, inefficiency, and low learning success [4], [5], [6]. Simply “letting students research’ tends to produce frustration rather than researchers.

Although Huber himself, for example together with Reinmann, later emphasized the need for process-supporting guidance by teachers (“scaffolding”) [7], the core terminology of this concept was never systematically adapted to this crucial insight. Moreover, there is a critical question as to whether the ideal of research-based learning primarily reflects a humanities and social science understanding of science – shaped by individual textual work and critical reflection – which is only partially compatible with the hypothesis- and team-based laboratory and clinical research in modern medicine. This lack of alignment with the disciplinary cultures of the life sciences is another reason why research-based learning – unlike internationally established Inquiry-Based Learning [8] – has hardly been adopted outside the German-speaking world. In a world where knowledge is ubiquitous and always accessible, the goal of education fundamentally shifts: competency development is not in opposition to education but its contemporary expression. It is time to transform the educational policy impulses of that era into a structured, practice-oriented, and scientifically grounded educational model for today.

**Figure 1:**
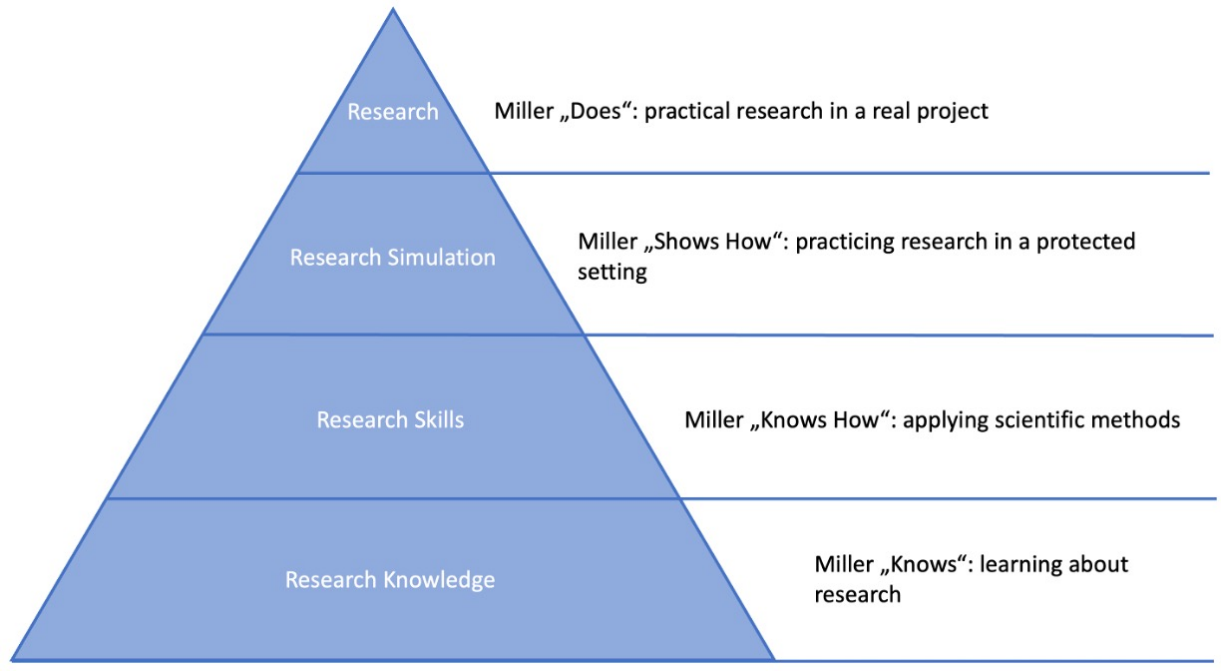
Representation of “research and learning” as a pyramid based on Miller [9]

Effective science-oriented teaching requires structured, practical, and empirically based learning concepts. A differentiated nomenclature of science-oriented learning formats has been lacking so far. This article proposes such a nomenclature by systematically categorizing learning formats according to competence acquisition, using Miller’s Pyramid as a guide [9]. Although aspects of these stages are already established in various teaching contexts, the key added value of this nomenclature lies in its coherent systematization. Linking learning formats progressively to the competence levels of Miller’s Pyramid creates a clear developmental logic for scientific competencies. This enables precise design and localization of educational offerings and directly maps competence development to the requirements of the NKLM chapter on scientific-medical competencies (see table 1 (Tab. 1) and figure 1 (Fig. 1)):

- **Level 1: research knowledge (knows: learning about research): **Absorption and processing of established theories, models, and key research findings. These formats (e.g., lectures) are highly structured and focus on building a solid foundation for subsequent levels.
- **Level 2: research skills (knows how: applying scientific methods): **Targeted application and practice of specific scientific methods and techniques. In moderately structured formats (e.g., seminars and medical practical courses), critical-logical thinking is promoted as students learn to correctly use research tools.
- **Level 3: research simulation (shows how: practicing research in a controlled environment): **Independent handling of a complete but didactically structured research process in a controlled setting. Here, the application of competencies is practiced through simulations (e.g., problem-based learning) or closely supervised projects to integrate methodological and practical skills under targeted guidance.
- **Level 4: research (does: conducting real research projects): **Active and largely independent participation in real research projects (e.g., in scientific projects as per the new licensing regulations or as part of a doctoral thesis) with authentic research interests. Here, process-supporting supervision (“scaffolding”) enables full integration into the research process and development of professional research competence.

International best practices such as inquiry-based learning [8], problem-based learning [10], and project-based learning [11] show that targeted guidance combined with practical research applications is particularly effective. Especially conducting real research projects, which actively involve students, has proven particularly sustainable [12].

The proposed nomenclature promotes a balanced combination of guidance and independence and offers significant advantages over existing models. Compared to the research-teaching nexus of Healey and Jenkins [13] – which differentiates between research-led, research-oriented, research-tutored, and research-based learning – the new nomenclature provides a clearer distinction and more practical differentiation between simulated and real research experiences. Compared to guided inquiry-based learning [8], which emphasizes structured guidance, this nomenclature offers a progressive structure and clear competence assignment, integrating the medical training logic and strongly supporting interdisciplinary and digital research tools in the respective learning stages. Crucially, it replaces open, unstructured formats with stepwise didactic framing, thus avoiding overload and arbitrariness in favor of verifiable, curriculum-compatible competence development.

At the same time, this nomenclature is particularly well-suited for analyzing existing curricula and precisely locating science-oriented learning formats. Its clear staging along an adapted Miller Pyramid for scientific competencies allows for precise localization and highlights each format's contribution to competence acquisition. Examples from the DACH region (Germany, Austria, Switzerland) illustrate this applicability:

The Faculty of Medicine at the University of Cologne realizes actual research (Level 4) through its “Research and Medical Studies” (FuM) program, actively involving students in the research process [14]. Hannover Medical School (MHH) emphasizes applying scientific methods (Level 2) through its longitudinal science module, characterized by interdisciplinary and practice-oriented teaching methods [15]. RWTH Aachen combines research simulation (Level 3) with aspects of actual research (Level 4) in its longitudinal scientific curriculum (LoWiCu) [16]. LMU Munich strengthens actual research (Level 4) through its “MeCuM Science” module, which promotes independent research projects [17]. At the University of Augsburg, the Scientific Longitudinal Course (WLK) primarily focuses on acquiring scientific foundations (Level 1) [18]. The Charité develops scientific competencies focused on method application (Level 2) through its Dieter Scheffner Center [19]. The University Medical Center Hamburg-Eppendorf (UKE) integrates elements of actual research (Level 4) through independent research projects [20]. The MHB Brandenburg also establishes actual research (Level 4) through a combination of method training and independent projects [21].

In summary, the nomenclature for science-oriented learning and teaching presented here provides a well-founded, practice-oriented, and empirically adaptable structure for designing curricular scientific competence development in medical education. By systematically structuring development along established stage models like Miller’s Pyramid, it creates a clear framework for designing, analyzing, and further developing medical curricula. The evaluation of practical examples from the DACH region confirms not only the applicability and relevance of this system but also highlights that structured and guided learning formats have long dominated good teaching practice – and for good reason. The nomenclature makes this development visible, categorizes it didactically, and enables targeted and sustainable promotion of research competence.

With its ability to integrate interdisciplinary perspectives, digital tools, and new competence fields such as Open Science or AI, it provides not only a practical framework but also a strategic building block for future-oriented, internationally compatible science-oriented education – especially in implementing NKLM 3.0. Scientific thinking is not an innate ability but a complex mental field of action – with its own rules, strategies, and typical pitfalls. It does not arise by itself but only through targeted guidance, systematic support, sufficient time for reflection, and joint engagement with one’s own research in exchange with others [22]. This is the didactic core of our nomenclature – and what distinguishes it from the often idealized notion of a self-unfolding researcher subject.

At the same time, this approach points beyond medical education to an educational policy responsibility: Those who do not systematically teach scientific thinking leave it to chance – with consequences that are already evident in an increasingly unbounded public knowledge space.

Despite the differentiation of science-oriented learning forms outlined here, there remains considerable research need to evaluate the effectiveness and sustainability of these approaches. Future work must clarify, through systematic studies, which differentiated learning forms achieve the greatest learning success in which context – both short-term and in terms of long-term scientific practice. Systematic evaluations, such as those carried out in research consortia focused on developing and testing specific competencies in innovative learning environments (e.g., DFG Research Group 2385 on promoting diagnostic competencies in simulation-based higher education learning settings), or as demanded and conducted by medical education experts, are essential.

Further, more detailed research is needed on the influence of structured feedback mechanisms and the promotion of self-regulation skills on learning success in research-oriented teaching formats [23]. Digital competencies, Open Science, and responsible AI use: Given the advancing digitalization of science, it is crucial to clarify how digital skills, use of digital tools, Open Science practices [24], and especially the development of a critical understanding and responsible use of AI tools – including generative AI – can be meaningfully integrated into science-oriented teaching, especially in medical education [25]. Another largely unexplored area concerns the long-term impact of science-oriented teaching on later professional practice, particularly regarding evidence-based practice and critical thinking [26]. Finally, comparisons with international perspectives and best practices and the transferability of successful models to the German-speaking context offer important potential for future research [27].

### This issue

This issue focuses on current developments, innovative formats, and research-based findings in medical education. It addresses curricular and structural questions as well as psychosocial, ecological, and didactic dimensions of medical training.

Theurich et al. [28] take a broad curricular view, systematically aligning the Berlin Model Curriculum with NKLM 2.0, showing increased but still insufficient coverage of competence goals – with implications for future revisions. Scheffer et al. [29] discuss the extracurricular yet highly relevant engagement of medical students during the COVID-19 pandemic, underscoring the potential of structured practice phases for clinical and social competence development.

The promotion of interactional and communication competencies is another focus. Schütte et al. [30] report on the IKM selection procedure used at Heidelberg University, which reliably measures applicants’ interactional skills. Laudage et al. [31] build on this, presenting an empirically based prioritization of communication content for medical education aligned with everyday medical practice relevance. Wellensiek et al. [32] add a nursing education perspective, showing that collegial consultation during nursing training supports professional identity development and mental well-being.

Ecological and psychosocial aspects are also increasingly entering curricula. Heinen et al. [33] present an innovative team-teaching seminar on the stigmatization of visible skin diseases, combining dermatological and psychosocial perspectives. Gebhardt et al. [34] address “eco emotions”, showing how psychotherapy training can be expanded to include dealing with climate-related emotions. Lilier et al. [35] report on “Klima-LIMETTE”, a student-developed course format on planetary health education with simulated patients, now integrated into the curriculum.

Didactic quality and its effect on educators are at the center of Kiver et al.’s [36] study: Teaching-related self-efficacy among young physicians correlates with motivation and satisfaction, strengthened by teaching experience and didactic training. González Blum et al. [37] analyze structural and legal factors affecting the sustainable establishment of interprofessional teaching and formulate conditions for success at medical faculties.

Innovative teaching and examination formats complete the issue: Scherff et al. [38] evaluate “EYE-ECG2”, an eye-tracking-based training video for ECG interpretation. Results show learning gains, especially among clinically experienced students. Finally, Spitznagel et al. [39] demonstrate, using an example of a workshop on stress management in emergency medicine, how targeted training formats can improve action confidence under pressure.

This diversity of topics and methodological approaches reflects the dynamic evolution of medical education – between curricular consolidation, social relevance, and individual competence development.

## Author’s ORCID

Christoph Stosch: [0000-0003-1001-4310]

## Competing interests

The authors declare that they have no competing interests. 

## Figures and Tables

**Table 1:**
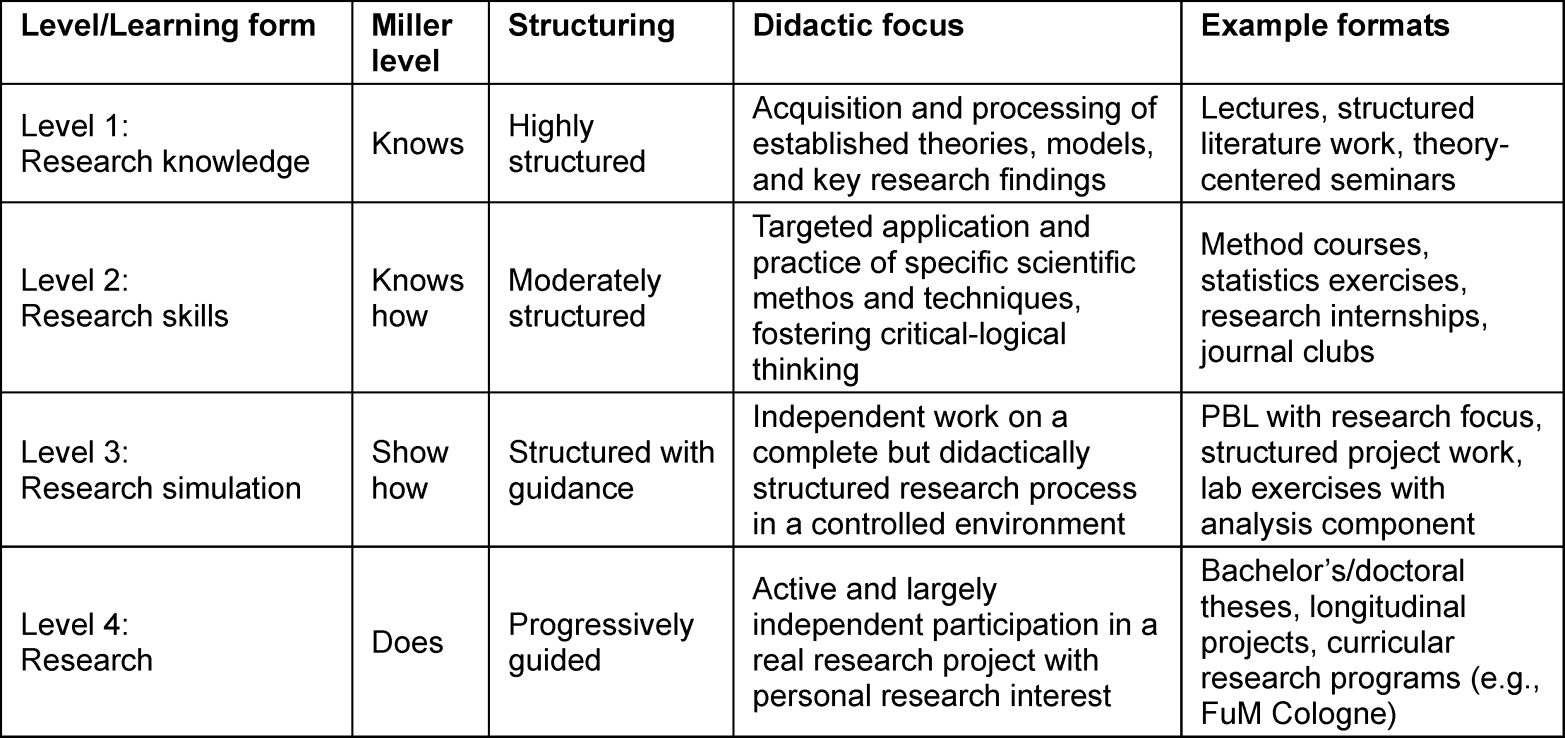
Systematic overview of the “research and learning” model
